# Preoperative assessment of tumor size by MRI and ultrasound in cervical cancer: a large-scale retrospective comparative study

**DOI:** 10.1007/s00404-026-08304-7

**Published:** 2026-01-09

**Authors:** Kaili Wang, Lulu Si, Mengling Zhao, Ruixia Guo

**Affiliations:** 1 Department of Gynecology, The First Affiliated Hospital of Zhengzhou University, Zhengzhou, 450000 Henan China; 2 Department of Gynecology, The Third Affiliated Hospital of Zhengzhou University, Zhengzhou, 450000 Henan China

**Keywords:** Preoperative tumor size, Magnetic resonance imaging, Ultrasound, Cervical cancer, Imaging modalities, Diagnostic accuracy

## Abstract

**Purpose:**

Accurate preoperative evaluation of tumor sizes is essential for guiding optimal treatment planning in cervical cancer. This study aimed to compare the accuracy of preoperative tumor size measurement between magnetic resonance imaging (MRI) and ultrasound.

**Methods:**

A retrospective study was performed involving 925 patients diagnosed with cervical cancer who underwent primary surgical treatment between January 2020 and June 2025. The accuracy of these two imaging modalities was assessed by comparing their measurements to the maximum tumor diameter determined through postoperative pathological analysis.

**Results:**

The Bland–Altman analysis showed that both ultrasound (mean difference: 1.50 mm) and MRI (mean difference: 0.61 mm) overestimated tumor size. In the paired subgroup of 757 patients who underwent both imaging modalities, the agreement rates between imaging and pathology for categorizing tumors into size groups were 65.8% for ultrasound and 67.6% for MRI (*p* = 0.360). Although MRI showed a significantly smaller mean measurement bias than ultrasound (0.73 mm vs. 1.37 mm; *p* = 0.012), the proportion of large errors (> 10 mm) was not significantly different. Multivariate analysis indicated that tumors > 40 mm assessed by ultrasound (OR = 2.85) or MRI (OR = 2.72) were significantly associated with increased likelihood of measurement discrepancies > 10 mm.

**Conclusion:**

While MRI exhibited a lower measurement error compared to ultrasound, both modalities showed comparable performance in tumor size staging. Furthermore, for tumors exceeding 40 mm in diameter as determined by preoperative imaging, clinicians are advised to integrate clinical examination to enhance the accuracy of staging.

**Supplementary Information:**

The online version contains supplementary material available at 10.1007/s00404-026-08304-7.

## What does this study add to the clinical work

**Table:** 

This large-scale retrospective study demonstrates that while MRI exhibited a lower measurement error compared to ultrasound, both modalities showed comparable performance in tumor size staging, supporting its viability as an alternative initial imaging modality, particularly in resource-limited settings. Furthermore, it identifies that a larger tumor size (> 40 mm) assessed preoperatively is an independent risk factor for significant measurement error with both ultrasound and MRI, highlighting the need for heightened caution when interpreting imaging findings for surgical planning in cases of bulky tumors.	

## Introduction

Cervical cancer remains a significant global health burden and a leading cause of cancer-related mortality among women in developing nations [1]. Given the significance of tumor size as a prognostic indicator for stage IB [2, 3], the FIGO 2018 staging system subdivides stage IB into three distinct categories based on tumor dimensions of 2 cm and 4 cm [4]. Radical hysterectomy with bilateral pelvic lymph node dissection represents the established surgical treatment for patients diagnosed with cervical cancer [5]. However, the ConCerv trial [6] and the recently published SHAPE trial [7] demonstrated that simple hysterectomy may be considered an alternative to radical hysterectomy for patients presenting with tumors smaller than 2 cm and limited depth of invasion. Precise tumor measurement guides surgeons in balancing oncologic control with quality of life considerations. It directly influences the selection of therapeutic strategies [5, 8].

The NCCN guidelines and the ESGO/ESTRO/ESP guidelines advocate for the use of pelvic magnetic resonance imaging (MRI) as the primary modality for preoperative assessment of the extent of local lesions in cervical cancer owing to its excellent soft-tissue resolution [5, 8–10]. The combination of diffusion-weighted imaging and T2-weighted imaging can further improve the detection of very small tumors and parametrial invasions [11, 12]. Concurrently, the emerging technology, such as fluorodeoxyglucose positron emission tomography/MRI, and the exploration of artificial intelligence promise a future of unprecedented precision in tumor characterization [11, 13]. However, the disease burden is disproportionately high in resource-limited regions where access to MRI is constrained, and advanced techniques like artificial intelligence are not yet available. Acknowledging this disparity, the 2023 update to the ESGO/ESTRO/ESP guidelines recognizes transvaginal/transrectal ultrasound, when performed by an expert sonographer, as an alternative in instances where MRI is inaccessible [14]. Ultrasound approaches are valued for their reliability in visualizing structures adjacent to pelvic organs, their ability to deliver high-resolution images of cervical tumors, real-time imaging capabilities, and cost-effectiveness, although their diagnostic accuracy may vary depending on the operator’s expertise [10, 15, 16].

While previous studies have established the value of both ultrasound and MRI [10, 17, 18], there remains a deficiency of large-scale investigations evaluating the accuracy of preoperative ultrasound and MRI in determining tumor size, the factors influencing the precision of tumor size assessment, and the impact of measurement errors on staging alterations. Therefore, we designed this large-scale retrospective study to compare the accuracy of preoperative tumor size measurement between MRI and ultrasound using postoperative histopathology as the reference standard, and to identify factors associated with measurement inaccuracy, thereby enhancing the reliability of preoperative staging for all patients.

## Materials and methods

### Study design and patient selection

This was a single-institution, retrospective cohort study. The study was approved by the Institutional Review Board of The First Affiliated Hospital of Zhengzhou University (Approval Number: 2025-KY-1420-001). Clinical data from patients diagnosed with cervical cancer and treated surgically at the First Affiliated Hospital of Zhengzhou University between January 2020 and June 2025 were collected. We used the FIGO 2018 system for staging cervical cancer, and preoperative clinical staging is determined through a combination of imaging tests and gynecological examination. The primary focus of this article is the accuracy of MRI and ultrasound in assessing tumor size. Only cases with detailed tumor size measurements reported from preoperative ultrasound and MRI were included. Exclusion criteria comprised preoperative neoadjuvant chemotherapy, lack of detailed tumor size measurement on preoperative imaging, and absence of pathological tumor diameter records. To ensure the relevance of preoperative measurements, only examinations performed within 14 days before the primary surgery were included in the analysis. Patients with cervical cancer confirmed through conization were also excluded due to concerns regarding the challenges with accurately measuring the postoperative pathological tumor diameter.

Three-dimensional transvaginal or transrectal ultrasound was performed utilizing endoluminal high-resolution probes at 5–9 MHz for imaging purposes. MRI was carried out with a 3.0 Tesla scanner using a pelvic phased-array coil. The imaging protocol uniformly included the following essential sequences for tumor localization and characterization: high-resolution T2-weighted imaging in three planes—sagittal, axial, and oblique (perpendicular and parallel to the long axis of the cervix); T1-weighted imaging in the axial plane; diffusion-weighted imaging in the axial or oblique axial plane; dynamic contrast-enhanced MRI performed after intravenous administration of a gadolinium-based contrast agent. Typical parameters: slice thickness of 3–4 mm with a minimal gap of 0.5–1 mm. The final pathological tumor size was determined by macroscopic and microscopic examination of the surgically resected specimen after routine formalin fixation (with a certain risk that the size will be underestimated due to tissue shrinkage). We selected the maximum tumor diameter as the parameter for comparative analysis. The sonographers and radiologists were blinded to the findings of the other imaging when generating the clinical reports, which were subsequently adhered to standardized departmental guidelines.

### Statistical analysis

Statistical analyses were conducted utilizing SPSS Statistics 26.0. The Shapiro–Wilk test was employed to assess the normality of the continuous variables. For measurement data that did not conform to a normal distribution, values were presented as the median (interquartile range (IQR)). Bland–Altman analysis was employed to characterize the measurement discrepancies between imaging modalities and pathological findings. Proportional bias and heteroscedasticity were tested using linear regression and the Breusch–Pagan test. The intraclass correlation coefficient (ICC) was calculated using a two-way random-effects model focused on absolute agreement, specifically using the single-rater ICC to represent the clinical scenario of one observer. ICC values for MRI and ultrasound were determined separately within their respective patient groups. Multivariable logistic regression models were developed for ultrasound and MRI to identify factors associated with a high risk of substantial measurement error. The findings are reported as adjusted odds ratios (OR) accompanied by their respective 95% confidence intervals (CI). For direct comparisons between ultrasound and MRI accuracy, we analyzed the paired subgroup of patients who underwent both imaging modalities with the Wilcoxon signed-rank test and McNemar’s test. A two-tailed p-value less than 0.05 was considered indicative of statistical significance.

## Results

**Fig. 1:**
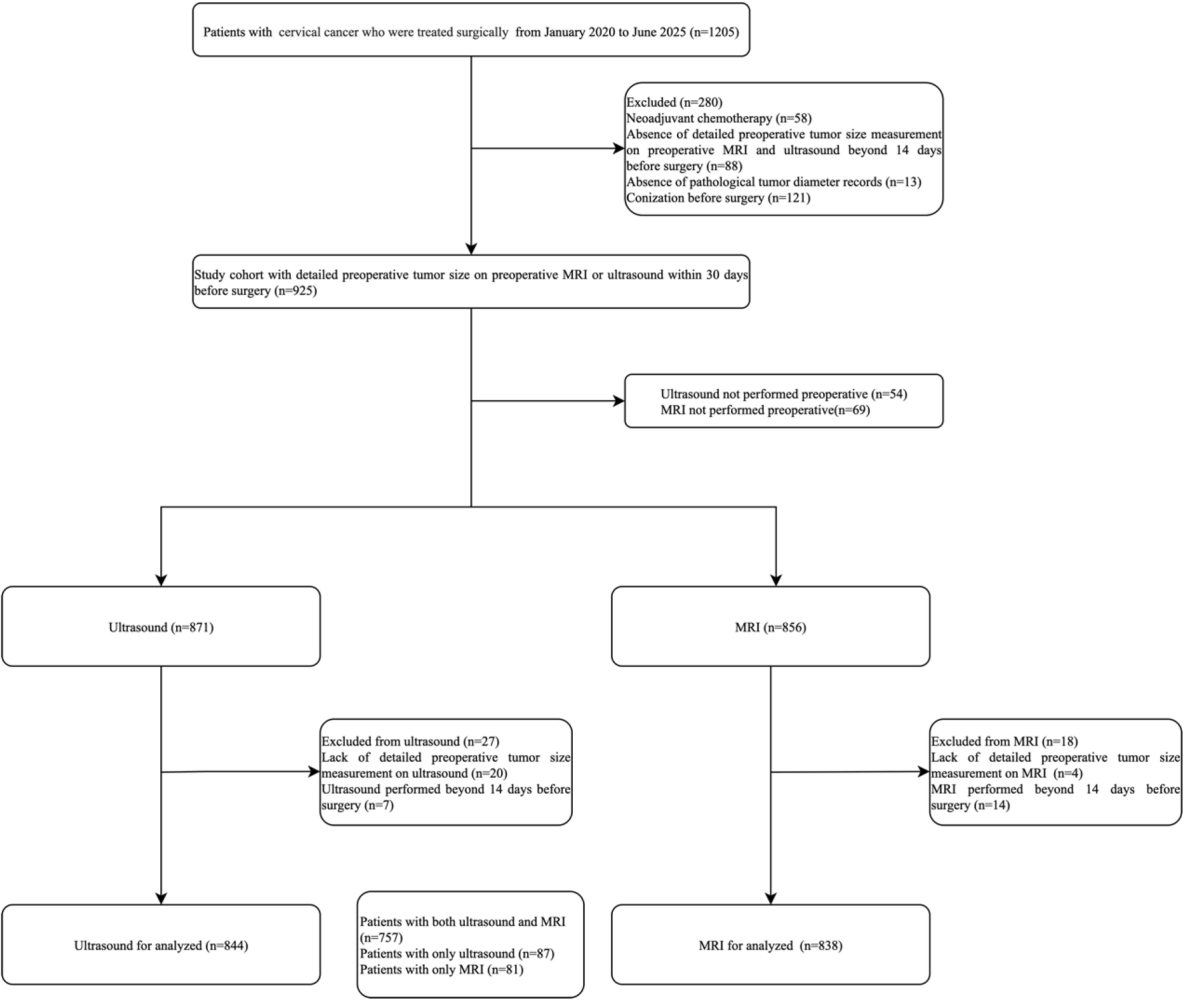
Flowchart of study population inclusion and exclusion

A total of 925 patients constituted the study cohort after excluding 280 patients who received neoadjuvant chemotherapy, lacked preoperative or pathological tumor size data, or underwent conization before surgery. Among these, 757 patients underwent both imaging modalities (Fig. 1).

Data extracted from electronic medical records included patient age, body mass index (BMI), menopausal status, FIGO stage, histological subtype, and final pathological examination, as well as the case of positive lymph node (Table 1). The population characteristics were similar between the ultrasound-only (*n* = 87) and MRI-only (*n* = 81) groups, with no significant differences (Supplementary Table 1).

**Table 1:** Baseline clinical and pathological characteristics of the patients

Characteristics	All patients (*n* = 925)
Median age (IQR), years	52 (43–58)
Median BMI (IQR), kg/m^2^	24.13 (22.03–26.56)
BMI category	
≤ 25 kg/m^2^	551 (59.6%)
25–30 kg/m^2^	314 (33.9%)
> 30 kg/m^2^	60 (6.5%)
Menopause	461 (49.8%)
Histotype	
Squamous	737 (79.7%)
Adenocarcinoma	136 (14.7%)
Adenosquamous	33 (3.6%)
Others	19 (2.1%)
Maximum tumor size (final pathology)	
≤ 20 mm	271 (29.3%)
20–40 mm	565 (61.1%)
> 40 mm	89 (9.6%)
LN-positive	185 (20%)

Results are presented as median (IQR) or frequency (percentage)

BMI: Body Mass Index. LN-positive: positive lymph nodes

**Fig. 2:**
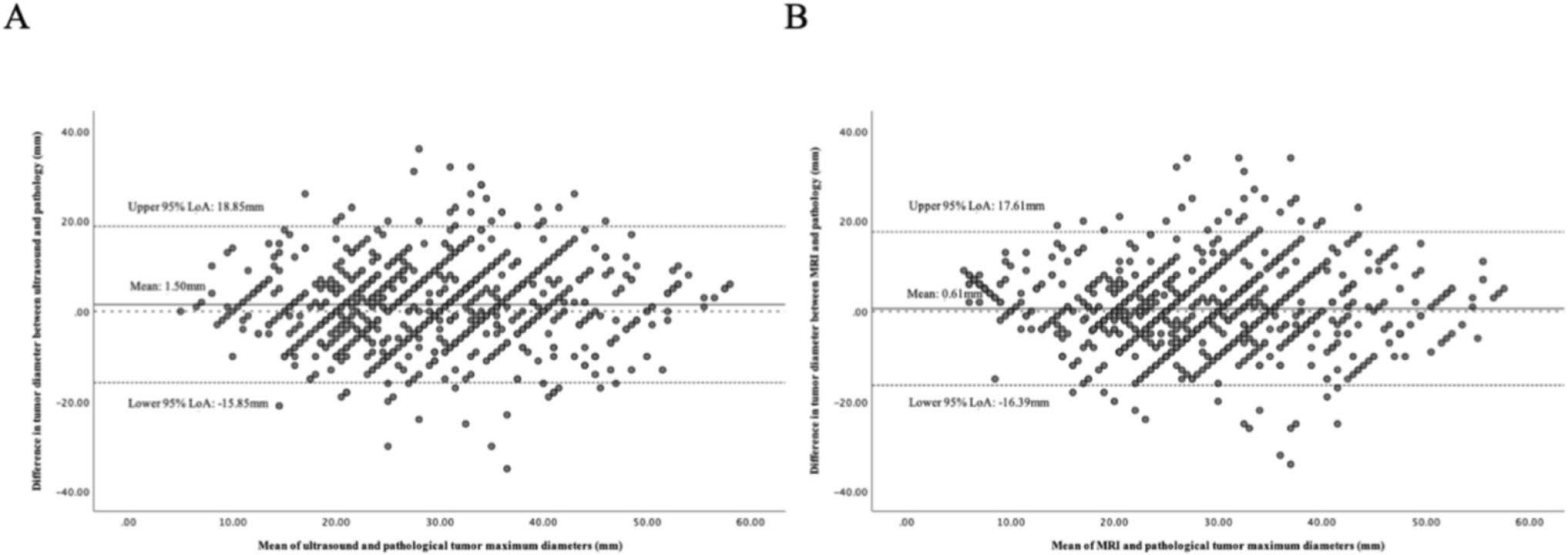
Bland–Altman plots of the discrepancy between ultrasound (**A**) and MRI (**B**) with postoperative pathological measurements. The Bland–Altman analysis showed that the mean raw discrepancy between the maximum tumor diameter measured by ultrasound and that measured by postoperative pathology was 1.50 ± 8.85 mm, with 95% limits of agreement (LoA) ranging from − 15.85 mm to 18.85 mm. The regression of the difference on the mean did not indicate any trend change as the tumor size increased (*β* = 0.034, *p* = 0.283). The Breusch–Pagan test indicated no evidence of heteroscedasticity (*p* = 0.803) (Fig. 2A). For MRI, the Bland–Altman analysis revealed a mean raw discrepancy of 0.61 ± 8.67 mm compared to postoperative pathology, with a 95% LoA spanning from − 16.39 mm to 17.61 mm

There was no significant proportional bias and heteroscedasticity observed for MRI (*p* > 0.05) (Fig. 2B).

The ICC between MRI and pathology was 0.70 (95% CI 0.66 to 0.73), while that between ultrasound and pathology was 0.65 (95% CI 0.60 to 0.70). The difference between the two ICC values was not statistically significant (*Z* = − 1.883, *p* = 0.059).

Multivariate logistic regression analysis revealed that, in comparison to tumors measuring ≤ 20 mm, those exceeding 40 mm were associated with a significantly higher likelihood of measurement errors greater than 10 mm when assessed by both ultrasound and MRI (ultrasound: OR = 2.85, 95% CI 1.71–4.77, *p* < 0.001; MRI: OR = 2.72, 95% CI 1.63–4.55, *p* < 0.001). Conversely, pathological type, menopausal status, BMI, ultrasound approach, time from imaging to surgery, and the number of vaginal deliveries did not demonstrate significant associations with substantial discrepancies in preoperative imaging measurements (Table 2).

**Table 2:** Preoperative variables correlated with discrepancies exceeding 10 mm in tumor size measurements during imaging assessments

Variable	Ultrasound (*n* = 844)		MRI (*n* = 838)	
	OR (95% CI)	*p*-value	OR (95% CI)	*p*-value
Histotype				
Squamous	Reference	–	Reference	–
Adenocarcinoma	0.94 (0.56–1. 56)	0.801	0.86 (0.51–1. 44)	0.571
Others	0.94 (0.45–1.99)	0.879	0.87 (0.40–1.88)	0.716
Preoperative tumor size				
≤ 20 mm	Reference	–	Reference	–
20–40 mm	0.91 (0.58–1.43)	0.684	0.75 (0.49–1.16)	0.200
> 40 mm	2.85 (1.71–4.77)	< 0.001*	2.72 (1.63–4.55)	< 0.001*
Ultrasound approach				
Transvaginal	Reference	–	–	–
Transrectal	1.72 (0.82–3.60)	0.153	–	–
Menopause				
No	Reference	–	Reference	–
Yes	0.87 (0.60–1.26)	0.473	0.83 (0.57–1.21)	0.322
BMI				
≤ 25	Reference	–	Reference	–
25–30	1.27 (0.88–1.85)	0.201	0.95 (0.64–1.40)	0.787
> 30	1.57 (0.78–3.15)	0.204	0.90 (0.41–1.95)	0.783
Number of vaginal births				
0	Reference	–	Reference	–
1	0.98 (0.52–1.86)	0.947	0.92 (0.48–1.76)	0.798
≥ 2	0.88 (0.51–1.52)	0.636	0.75 (0.43–1.31)	0.309
Time from imaging to surgery	1.04 (0.93–1.16)	0.511	0.98 (0.87–1.10)	0.711

*OR* odds ratios; *CI* confidence intervals

*Stands for *p* < 0.05

In 757 patients who underwent both imaging modalities, the agreement rates between preoperative ultrasound and MRI measurements of tumor size were 65.8% and 67.6%, based on postoperative pathological tumor diameters of 20 mm and 40 mm, with no statistically significant difference observed (*p* = 0.360) (Table 3).

**Table 3:** The agreement rate in categorizing tumors into size groups (≤ 20 mm, 20–40 mm, > 40 mm) between imaging and pathology

A. Ultrasound vs. Pathology					
Maximum Tumor size(mm)		Ultrasound			Agreement (%)
		≤ 20	20–40	> 40	
Pathology	≤ 20	61	75	9	65.8%
	20–40	63	379	81	
	> 40	3	28	58	

B. MRI vs. Pathology					
Maximum Tumor size(mm)		MRI			Agreement (%)
		≤ 20	20–40	> 40	
Pathology	≤ 20	72	66	7	67.6%
	20–40	71	388	64	
	> 40	4	33	52	

In 757 patients who underwent both imaging modalities, the mean raw discrepancy between the maximum tumor diameter measured by ultrasound and MRI compared to that determined by postoperative pathology was 1.37 ± 10.14 mm and 0.73 ± 8.73 mm, respectively, with this difference reaching statistical significance (*p* = 0.012). The absolute differences for ultrasound and MRI were 7.57 ± 6.84 mm and 6.99 ± 7.05 mm, respectively, and these differences were not statistically significant (*p* = 0.123). The proportion of cases exhibiting discrepancies greater than 10 mm was comparable between MRI (20.5%) and ultrasound (22.1%) (*p* = 0.369) (Table 4).

**Table 4:** The discrepancy in maximum tumor diameter measurements between ultrasound and MRI relative to postoperative pathological assessment

Discrepancy size (*n* = 757)	Ultrasound	MRI	*Z*/*χ*^2^	*p*
Raw differences (mm)	1.37 ± 10.14	0.73 ± 8.73	− 2.508	0.012*
Absolute differences (mm)	7.57 ± 6.84	6.99 ± 7.05	− 1.541	0.123
Discrepancy size over 10 mm	167 (22.1%)	155 (20.5%)	0.807	0.369

Raw differences were calculated as imaging measurement—pathological measurement, indicating the direction of bias (positive value = overestimation, negative value = underestimation). Absolute differences were the absolute value of the raw differences, representing the magnitude of the measurement error regardless of direction

*Stands for *p* < 0.05

The staging changed in 30.3% of cases when comparing the preoperative assessment to the postoperative pathological staging. Changes in staging were observed in 171 cases (18.5%) due to errors in measuring tumor diameter, and 122 cases (13.2%) because of incomplete evaluation of vaginal involvement. A Sankey diagram visually depicts the flow of patients between preoperative clinical stages and postoperative pathological stages (Supplementary Fig. 1). The most common stage transitions in the stage change caused by tumor size were from preoperative IB1 to postoperative IB2, representing 18.6% (48/258) in the preoperative IB1 group.

If the criteria for surgical radicality are a tumor size over 20 mm, and chemoradiation is used for tumors larger than 40 mm, then 159 cases (17.2%) would have been managed incorrectly, with the most frequent management change being that 5.6% would have undergone an overly radical procedure (Table 5).

**Table 5:** Potential Impact of Tumor Size Measurement Errors on Clinical Management

Staging change	Management change			
	Under-treatment	Over-treatment	Incorrect primary surgery	Incorrect primary chemoradiation
171 (18.5%)	48 (5.2%)	52 (5.6%)	32 (3.5%)	27 (2.9%)

Under-treatment: a simple hysterectomy is performed for a tumor requiring a radical hysterectomy. Over-treatment: a radical hysterectomy is done for a tumor that only needs a simple hysterectomy or a fertility-preserving procedure. Incorrect primary surgery: performing surgery on a tumor that should be treated with chemoradiation. Incorrect primary chemoradiation: administering chemoradiation to a tumor that should be treated with surgery.

## Discussion

The findings of the current study indicate that while MRI exhibited a lower measurement error compared to ultrasound, both modalities showed comparable performance in tumor size staging. Furthermore, in tumors with a preoperative assessed diameter greater than 40 mm, the probability of measurement inaccuracies exceeding 10 mm markedly increases across both imaging techniques. Approximately 20% of staging alterations can be attributed to inaccuracies in the preoperative evaluation of tumor size in our study, which underscores the limitations in contemporary clinical methodologies for the precise evaluation of tumor dimensions.

The NCCN guidelines identify a tumor size threshold of 20 mm as suitable for fertility-preserving interventions. Evidence from a large multicenter retrospective study supports the safety of cervical surgery with fertility preservation in patients presenting with tumors measuring 20 mm or less. In contrast, tumors exceeding 20 mm in diameter are associated with an elevated risk of recurrence [19]. For patients who do not require fertility preservation, the SHAPE and ConCerv clinical trial suggest that a simple hysterectomy may be an appropriate alternative to radical hysterectomy for individuals with low-risk cervical cancer measuring less than 20 mm. If the tumor diameter is underestimated before surgery and a simple hysterectomy is performed, there may be a requirement for additional surgical intervention or supplementary pelvic external radiation therapy due to the inadequate extent of resection achieved in the initial operation. In the SHAPE study, there were only 2.5% of patients upstaged due to the tumor’s size exceeding 20 mm [7]. The lower proportion compared to our study may be attributed to the selection of patients with smaller tumors. Concurrent chemoradiotherapy is preferred for cases involving large tumors greater than 40 mm in diameter. However, it has been reported to cause more severe pain during sex and urinary symptoms [20]. Underestimation of tumors larger than 4 cm may lead to primary surgical treatment, which is frequently followed by the necessity for adjuvant radiotherapy and/or chemotherapy. A recent post hoc analysis from the SNTIX trial on the accuracy of preoperative tumor size assessment in IA1-IB2 showed that 11.2% of patients would receive insufficiently radical surgery, while 6.5% would undergo unnecessarily radical procedures when the threshold is set at a tumor size of 20 mm [21]. In our findings, 5.2% of patients would receive inadequately radical surgery because of preoperative underestimation of tumor size, and 5.6% of patients would undergo unnecessarily radical procedures as a result of overestimation. The elevated incidence of insufficient surgical intervention observed in their study can be attributed to the greater proportion of patients presenting with tumors smaller than 20 mm in their study population. Since their study subjects were tumors within 40 mm, the 40 mm threshold was not established to evaluate the error rate in the initial surgery or chemoradiotherapy choice. In our study, we used the 20 mm and 40 mm thresholds and showed that 159 cases (17.2%) would have been managed incorrectly.

Both ultrasound and MRI demonstrated a slight overestimation of tumor size, with mean raw differences of 1.50 ± 8.85 mm and 0.61 ± 8.67 mm. Pan et al. also found through Bland–Altman analysis that MRI and ultrasound examinations tend to systematically overestimate tumor size during preoperative assessment [22]. The significant difference observed in the raw measurements indicates that ultrasound tends to overestimate tumor size to a greater extent than MRI in our study. Fischerova et al., involving 95 patients with cervical cancer scheduled for surgical intervention, demonstrated a stronger correlation with tumor size as determined by pathological assessment compared to magnetic resonance imaging (ultrasound: *r* = 0.996; MRI: *r* = 0.980) [23]. Although this finding may appear contradictory, their reliance on correlation coefficients, which, while indicating proportional variation between measurements, do not necessarily reflect numerical agreement. For instance, although the discrepancy between ultrasound and pathological measurements of tumor size consistently remains at 10 mm, the correlation coefficient may still attain a value of 1. In contrast, the Bland–Altman analysis employed in our study provides more precise assessments of the actual differences [24]. The similar concordance between ultrasound and MRI in classifying tumors into size categories (≤ 20 mm, 20–40 mm, > 40 mm), along with similar ICC values, indicates that both imaging modalities demonstrate comparable efficacy in tumor size staging. Borčinová et al. analyzed the SNTIX trial showed that the choice of imaging modality (ultrasound vs. MRI) did not have a significant impact on tumor size differences [21]. Our findings of the raw discrepancies between ultrasound and MRI measurements differ from this. One explanation for this discrepancy is the inclusion of stage IA cases in the SNTIX trial. For stage IA and some IB1 cases, the resolution of ultrasound and MRI is limited, often making it impossible to identify the tumor or only indicating abnormal cervical mucosal signals or localized blood flow changes without precise tumor size measurements [16, 17]. Including these cases increases measurement errors, which leads to different results. The magnitude of error increases concomitantly with tumor size in the SNTIX trial. This finding aligns with the results of our study. Our multivariate analysis identified that a preoperative tumor size exceeding 40 mm constitutes a critical determinant influencing measurement accuracy. These results underscore the necessity for cautious interpretation of ultrasound and MRI assessments in cases involving large tumors.

While this study focused on the accuracy of tumor size measurement, a thorough preoperative evaluation should also assess parametrial invasion, lymph node involvement, and the extent of cervical stromal invasion, since these are crucial for deciding treatment options and predicting the prognosis. For the assessment of lymph node status in cervical cancer, Fruhauf et al. reported that the sensitivity and specificity of transrectal ultrasound assessed per patient to detect pelvic node macrometastases were 72.2% and 94.0%, respectively, showing similar diagnostic accuracy as reported for MRI [25]. However, surgical-pathological staging of pelvic lymph nodes is the method of choice in the early stages [16]. Although ultrasound and MRI imaging can offer valuable data regarding the dimensions and local invasion of tumors [11, 12, 16, 17], the evaluation of parametrial infiltration should be combined with a gynecological examination. A study by Sodeikat et al. demonstrated that the specificity of parametrial invasion assessed by MRI in tumors ≥ 25 mm was reduced. Clinical examination performed under general anesthesia exhibited significantly higher accuracy in identifying parametrial invasion compared to MRI alone (83% versus 76%, *p* = 0.003) [26]. Therefore, in cases of large cervical tumors where imaging measurements are prone to error, integrating findings from a clinical examination with imaging results could mitigate the risk of staging inaccuracies and guide more appropriate treatment decisions.

This study’s principal strength lies in its substantial sample size (*n* = 925), which afforded adequate statistical power. Nonetheless, several limitations warrant consideration. Firstly, the retrospective nature of the study introduces inherent risks of selection biases. Secondly, our study did not focus on systematically evaluating other factors for staging and prognosis, such as parametrial invasion and lymph node status. While tumor size is critical for FIGO staging and treatment decisions, it represents only one component of a comprehensive preoperative evaluation. Furthermore, although our imaging protocols were standardized, the involvement of multiple operators in both image acquisition and interpretation introduces inter-observer variability that was not quantifiable in this study. This limitation, while representative of clinical practice, may impede the reproducibility of our measurements. Finally, the generalizability of our findings is influenced by our study’s context, within a single and high-volume tertiary center. The operator-dependent nature of ultrasound and the potential for variation in equipment and imaging protocols for both modalities mean that performance may differ in other environments, particularly those without specialized training.

This study demonstrates that both MRI and ultrasound provide clinically comparable accuracy for preoperative tumor size assessment in cervical cancer and show comparable performance in tumor size staging. This supports the use of ultrasound by an expert sonographer as a viable alternative in settings where MRI access is limited. However, MRI showed an advantage over ultrasound in terms of raw bias, which suggests that MRI remains the more precise modality. Furthermore, clinicians should exercise heightened caution when interpreting imaging-based tumor size measurements for surgical planning in cases involving large cervical tumors. Future research should prospectively investigate strategies to enhance the accuracy of imaging assessments for large tumors, potentially through technical refinements or the integration of artificial intelligence methodologies.

## Supplementary Information

Below is the link to the electronic supplementary material.

Supplementary file1 (PDF 164 KB) Supplementary Table S1. Comparative analysis of the baseline clinical and pathological characteristics between the ultrasound-only (*n* = 87) and MRI-only (*n* = 81) groups. The results showed no statistically significant differences in age, BMI, menopausal status, histologic type, distribution of pathological tumor size (≤ 20 mm, 20–40 mm, >40 mm), or lymph node status (all *P* > 0.05). Supplementary Figure 1: A Sankey diagram visually depicts the flow of patients between preoperative clinical stages and postoperative pathological stages. Preoperative staging is shown on the left side, whereas postoperative pathological staging is illustrated on the right side. The most common stage transitions in the stage change caused by tumor size were from preoperative IB1 to postoperative IB2, representing 18.6% (48/258) in the preoperative IB1 group.

## Data Availability

The data supporting the findings of this study are available upon reasonable request from the corresponding author.
