# Supplementary material for: Preoperative assessment of tumor size by MRI and ultrasound in cervical cancer: a large-scale retrospective comparative study
Source: Arch Gynecol Obstet. 2026 Jan 9;313(1):23. doi: 10.1007/s00404-026-08304-7 (PMC12789128; doi:10.1007/s00404-026-08304-7)

Preoperative Assessment of Tumor Size by MRI and Ultrasound in Cervical Cancer: A Large-Scale Retrospective Comparative Study

Journal name: Archives of Gynecology and Obstetrics

Kaili Wang, Lulu Si, Mengling Zhao, Ruixia Guo

Corresponding author: Ruixia Guo

Department of Gynecology, The First Affiliated Hospital of Zhengzhou University, Zhengzhou City, Henan Province, China, 450000.

E-mail address: grxcdxzzu@163.com.

Supplementary Table 1: The characteristics between the ultrasound-only (n=87) and MRI-only (n=81) groups

Characteristics	Ultrasound (n=87)	MRI (n=81)	P-value
Mean age (SD), years	51.26±1.05	50.07±1.18	0.452
Median BMI (IQR), kg/m2	23.44(21.56-25.87)	23.83(21.48-25.39)	0.987
Menopause	47(54.0%)	33(40.7%)	0.092
Histotype			0.069
Squamous	72(82.8%)	56(69.1%)	
Adenocarcinoma	10(11.5%)	21(25.9%)	
Adenosquamous	4(4.6%)	2(2.5%)	
Others	1(1.1%)	2 (2.5%)	
Maximum tumor size (final pathology)			0.089
≤20 mm	60(69.0%)	66 (81.5%)	
20-40 mm	26(29.9%)	15(18.5%)	
>40 mm	1(1.1%)	0	
LN-positive	11(12.6%)	6(7.4%)	0.482

Supplementary Figure 1: The flow between preoperative clinical stages and postoperative pathological stages

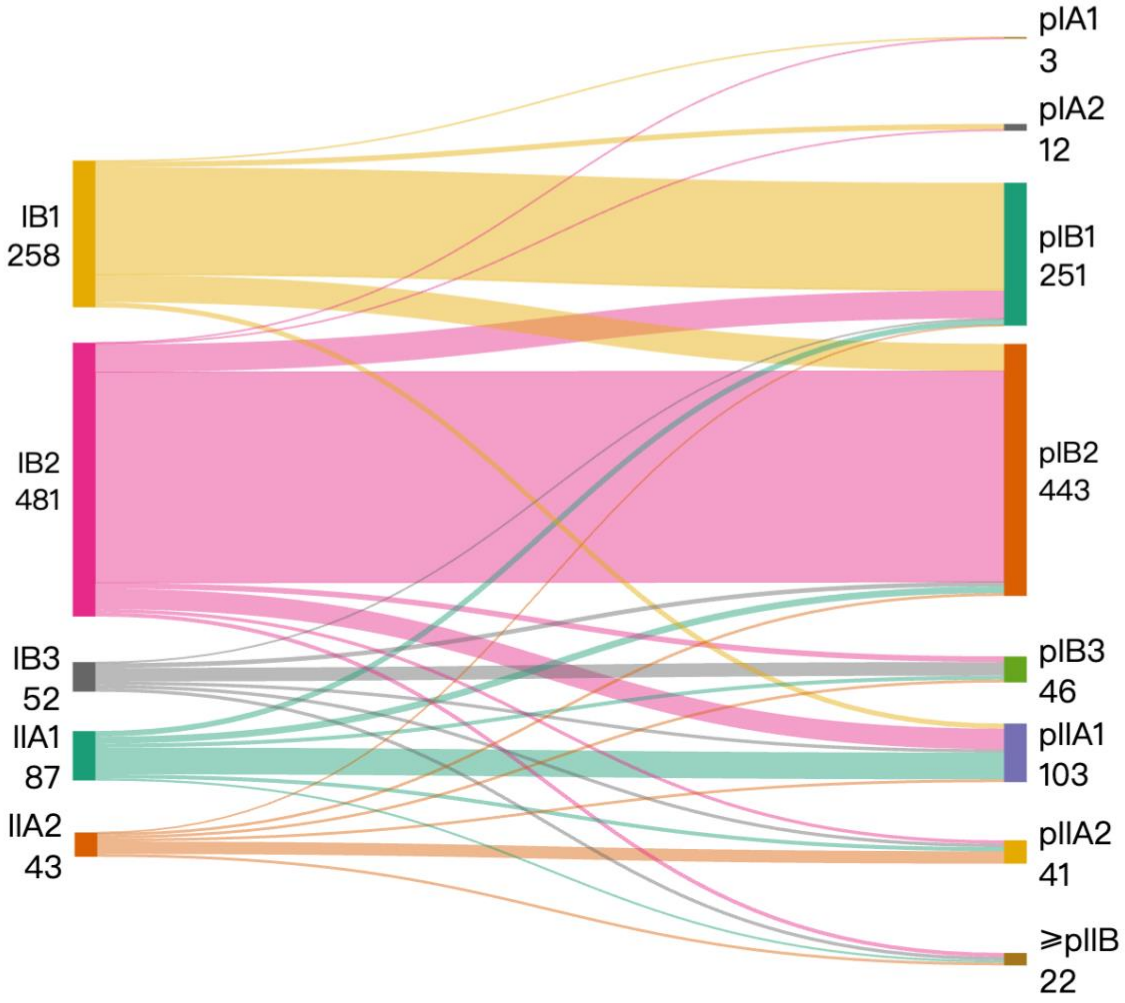

Supplement: Supplementary file 1 — Supplementary file1 (PDF 164 KB) Supplementary Table S1. Comparative analysis of the baseline clinical and pathological characteristics between the ultrasound-only (n = 87) and MRI-only (n = 81) groups. The results showed no statistically significant differences in age, BMI, menopausal status, histologic type, distribution of pathological tumor size (≤ 20 mm, 20–40 mm, >40 mm), or lymph node status (all P > 0.05). Supplementary Figure 1: A Sankey diagram visually depicts the flow of patients between preoperative clinical stages and postoperative pathological stages. Preoperative staging is shown on the left side, whereas postoperative pathological staging is illustrated on the right side. The most common stage transitions in the stage change caused by tumor size were from preoperative IB1 to postoperative IB2, representing 18.6% (48/258) in the preoperative IB1 group. [file 404_2026_8304_MOESM1_ESM.pdf]
